# Progress in Surgery

**Published:** 1897-05-29

**Authors:** 


### Progress in Surgery.

### BRAIN SURGERY.

Judging from recent discussions and from papers which have appeared within the last few months, especially in America, the present opinion seems to be that cranial surgery has meanwhile ceased to advance. "At the present time it appears as if but a small percentage of patients who suffer from cerebral disease can be relieved by surgical interference," says one reviewer. The outcome of a study of reported cases, successful and otherwise, seems to bring out the fact that our ultimate prognosis for operations must be less favourable than it has been hitherto. The reasons for this are not far to seek. There is first the extreme difficulty in diagnosis, not only as to the nature of a given lesion, but also as to its site, and this in spite of the great advances which have been made in cerebral topography and localisation. In addition to this, the necessarily limited access, the exceedingly delicate nature of the brain, both physically and physiologically, the great uncertainty of complete removal of new formations, such as tuberculous collections or malignant growths; and, lastly, the risk, almost amounting to certainty, of leaving a lesion, in the form of a cicatrix, which may give rise to symptoms —all these factors tend to tell against satisfactory surgical statistics.

We shall take occasion later to particularise in reviewing individual papers.

**Cerebral Localisation.**—Much attention has recently been devoted towards improving and simplifying the operative technique. A new method for localising brain lesions has lately been described, with illustrations, by R. H. Cox,[1] who claims for it that it is applicable to skulls of all sizes and shapes, and that measurements in inches and the use of particular angles are unnecessary. His instrument consists of the mechanical device known technically as "lazy-tongs" formed into a circle, with two accessory loops. On the segments of one of these accessory loops are the numerals, beginning at each end with 1 and ending at the middle with 10; the other loop has its segments lettered from A to V. The circular part of the instrument is applied round the head, and the loops adjusted. On comparing the reading they give with a chart-map of the skull and brain, which is furnished with the instrument, accurate localisation of any definite area is obtained. The apparatus can best be understood by referring to the illustrations which accompany Cox's paper.

A much simpler and equally accurate method, requiring no apparatus beyond a measuring tape, and enjoying all the advantages of this one, was described by Professor Chiene, of Edinburgh, and referred to in a recent number of The Hospital.

**The Technique of Temporary Resection of the Skull.**—In a paper read before the New York Surgical Society,[2] Van Arsdale describes the various methods which have from time to time been employed for opening the skull. He believes that at present the principle most favoured is that of temporary osteoplastic resection of a large bone flap, the flap having the pericranium and integument attached to it at its base, which should contain the main blood supply of the parts. There are two ways of making such flaps, (1) by means of the chisel and mallet, and (2) by means of variously formed saws or drills, worked by a surgical engine or electric motor. Objections, dating back as far as Galen, 200 A.D., have been urged against the chisel and mallet for this purpose, and these are shared in by all the modern authorities. It is said to produce symptoms of cerebral concussion, sometimes necessitating the stoppage of the operation and resort to other means. It is necessarily a slow procedure; and it leaves a wide groove in the bone, which prevents accurate apposition when replacing the flap.

Of the saws used for opening the skull the simplest is Hey's, but most modern operators prefer one of the circular saws worked by a motor of some sort or other, which enables the work to be done much more rapidly. Much ingenuity has been exhibited in devising saws, drills, &c., to combine rapidity with safety, but without apparent corresponding practical advantage. Arsdale adds to the number of these circular-saws one which, he claims, achieves the hitherto impossible feat of sawing round a corner. In shape it is like an open umbrella, the tips of the ribs representing the teeth of the saw, while the rotation is communicated by a surgical engine to the ferrule. This saw is fitted into a handle terminating in two prongs, and to this handle is also fitted a guard which passes between the dura and the bone, so protecting the former from damage by the saw. A preliminary small trephine opening is necessary for the introduction of this guard, and for this purpose the writer has invented a trephine, which makes a very narrow opening in the bone. The advantages claimed for this instrument are—its capacity to saw in a circle; its safety and rapidity of action; it cuts a narrow and bevelled groove which facilitates replacement of the bone flap; acting from within outwards it adapts itself to any varying thickness of skull.

Erwin Payr[3] describes a series of instruments he has devised for making exploratory punctures into the brain. Holes are bored in the skull by means of drills of different sizes, and through these various instruments—syringes, forceps, probes, needles, &c.—are introduced for diagnostic purposes.

**Hæmorrhage in Brain Surgery.**—Much of the difficulty in removing cerebral tumour lies in the profuse hæmorrhage which is met with in these cases. Keen[4] calls attention to the best methods at our disposal for control of hæmorrhage under these circumstances. He finds Horsley's antiseptic wax satisfactory for arresting bleeding from the diploë. As regards the dura, if it be found necessary to ligature a vessel in its continuity, this is best done by passing a small semi-circular Hagedorn needle, armed with fine silk or catgut, round it, care being taken to avoid injury of the subjacent brain and cerebral veins. When an artery bleeds in the divided dura it may be seized with forceps and ligatured; veins are best secured by an armed needle being passed round them, as the forceps are apt to lacerate and tear the vessel. This is also true of cerebral veins.

**Puncture of the Lateral Ventricle.**—Three cases in which marked benefit has followed puncture of the lateral ventricle are reported by Von Beck.[5] (1) A boy of fourteen, who had four years previously been cured of middle ear disease ensuing on diphtheria, was suddenly attacked with great pain in the ear and right side of head, vomiting and coma, without fever. There was neuro-retinitis, stiffness of the neck, general hyperæsthesia, and a slow pulse, 54. On operating, the mastoid was found sclerosed, and the cells contained turbid serum; the lateral sinus was healthy; puncture of the temporal lobe of the brain was negative. The lateral ventricle was tapped, and seven drachms of cerebo-spinal fluid drawn off. This was followed by marked improvement. The pulse rose to 80, the patient became sensible, and the neuro-retinitis disappeared. On two subsequent occasions symptoms reappeared, but were promptly checked by the withdrawal of two and a half ounces of clear cerebro-spinal fluid from the lateral ventricle. For two years before reporting the boy has been in good health. (2) A boy of four, three weeks after a fracture of the frontal bone, suffered from meningitis, evidently acute and septic. The evacuation of an abscess in the region of the fracture gave but temporary relief, and the patient was *in extremis* fifteen days later. Two and a half ounces of turbid cerebro-spinal fluid were withdrawn, and the patient recovered rapidly and completely. (3) The third case was a girl aged thirteen, who suddenly became unconscious, but the insensibility soon passed off, and the patient complained of great pain in the head. These attacks became very frequent, and were accompanied by vertigo and vomiting, and subsequently vision became affected. Cerebral tumour was diagnosed. For the relief of intra-cranial tension the lateral ventricle was tapped, and the withdrawal of two and a half ounces of fluid gave temporary relief. On the reappearance of symptoms tapping was repeated, again with satisfactory results. She is still under observation.

[1] Lancet, Jan. 2, 1897. [2] Annals of Surgery, Oct., 1896. [3] Centralbl. f. Chirurg., Aug., 1896. [4] Internat. Med. Mag., 1896. [5] La Tribune Médicale, No. 13, 1896.

